# Completely Intracorporeal Robotic-Assisted Laparoscopic Ileovesicostomy

**DOI:** 10.1155/2014/823813

**Published:** 2014-01-29

**Authors:** MaryEllen T. Dolat, Greg Wade, B. Mayer Grob, Lance J. Hampton, Adam P. Klausner

**Affiliations:** Department of Surgery, Division of Urology, Virginia Commonwealth University School of Medicine, Richmond, VA 23298-0118, USA

## Abstract

We present a report of a completely intracorporeal robotic-assisted laparoscopic ileovesicostomy with long term follow-up. The patient was a 55-year-old man with paraplegia secondary to tropical spastic paresis resulting neurogenic bladder dysfunction. The procedure was performed using a da Vinci Surgical system (Intuitive Surgical, Sunnyvale, CA) and took 330 minutes with an estimated blood loss of 100 mL. The patient recovered without perioperative complications. He continues to have low pressure drainage without urethral incontinence over two years postoperatively.

## 1. Introduction

Originally described in 1955, ileovesicostomy is a surgical procedure performed as an alternative to conservative medical management for patients with neurogenic bladder dysfunction. In an ileovesicostomy procedure, a segment of ileum is anastomosed to the native bladder, creating a low pressure urinary storage and drainage system designed to help prevent renal damage that is associated with high intravesical pressures. Ileovesicostomy can lead to an overall reduction in morbidity by reducing urinary tract infections, preventing renal damage, and giving patients freedom from the burden of intermittent catheterization [1–5]. With the advent of minimally invasive surgery and its subsequent adaptation to ileovesicostomy, intraoperative blood loss, postoperative morbidity, length of hospital stay, and postoperative pain have been reduced [6]. In addition, intracorporeal anastomosis of the bowel after isolation of adequate ileal length has been hypothesized to further reduce the risk of incisional hernia, mesenteric thrombosis and ischemia, and postoperative ileus [7]. Two prior reports of completely intracorporeal robotic-assisted laparoscopic ileovesicostomy, including our own, have been presented as technique videos at urologic meetings (refs); however, this is the first case that includes long term patient follow-up [8, 9].

## 2. Case Presentation

A 55-year-old white man with paraplegia secondary to tropical spastic paresis resulting neurogenic bladder dysfunction presented to Urology Clinic with urodynamic studies demonstrating high pressure neurogenic detrusor overactivity (180 cm H_2_O). High pressures were documented despite an 18-month period of compliance with a regimen consisting of oral antimuscarinic therapy, fluid restriction, and intermittent self-catheterization. Relevant surgical history included ruptured appendix/peritonitis followed by appendectomy and bowel surgery and intrathecal baclofen pump placement in the left upper quadrant. He was referred for management of his high pressure neurogenic bladder and elected to proceed with a robotic-assisted laparoscopic ileovesicostomy.

### 2.1. Surgical Technique

**Figure 1:**
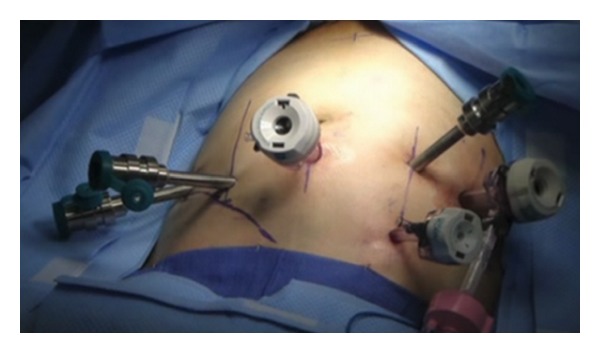
Intraoperative photograph of port placements.

**Figure 2:**
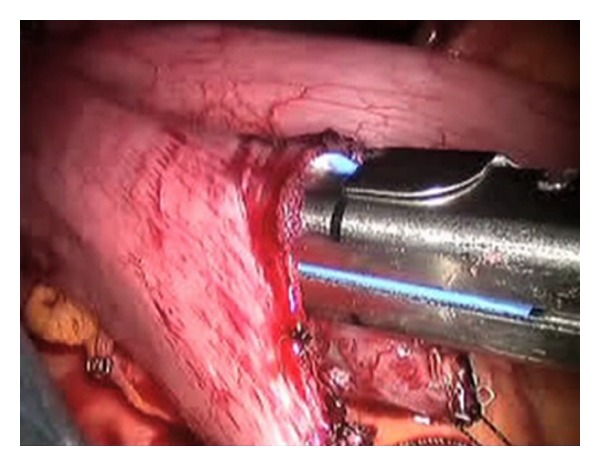
Intraoperative photograph of the intracorporeal bowel-to-bowel anastomosis using a 60 mm Endo GIA stapler.

The da Vinci Surgical system (Intuitive Surgical, Sunnyvale, CA) was used to perform the entire procedure. A video of the entire surgical procedure is available online at http://www.youtube.com/watch?v=QLBzoUEWVIg. The patient was placed in the dorsal lithotomy position in steep Trendelenburg at the start of the case. An 18-French foley catheter was placed. A Veress needle was introduced into the peritoneal cavity and insufflation was initiated to raise the intra-abdominal pressure to 15 mm Hg. Three robotic ports and two assistant ports (12 mm and 5 mm) were used in similar positions to that of a robotic-assisted laparoscopic prostatectomy (Figure 1) and the robot was docked. Adhesions in the right lower quadrant were taken down using electrocautery and the Ligasure device (Covidien, Boulder, CO). The ileocecal valve and ileum were then identified. A 3-0 silk suture holding stitch was placed in the ileum approximately 15 cm proximal to the ileocecal valve and another 3-0 silk marking stitch was placed approximately 15 cm proximal to the holding stitch. This ensured adequate length for an ileal loop from the top of the bladder to the future ostomy location. The mesentery for this demarcated section of ileum was opened proximally and distally, and the laparoscopic Endo GIA stapler (Ethicon, Somerville, NJ) was used to isolate a loop of ileum. Loose ends of the bowel were then reanastomosed by firing a 60 mm Endo GIA across the antimesenteric borders (Figure 2). A second staple was used to close the loop of bowel in a side-to-side fashion with minimal bowel spillage. A U-shaped bladder flap was then created using electrocautery. The proximal ileal loop was anastomosed to the bladder with running absorbable suture after the antimesenteric side was spatulated. Bladder irrigation was performed with no evidence of leak. A single stitch was placed on the distal end of the ileal loop which was then brought out through one of the ports. A 15-French round Jackson-Pratt drain was also placed through one of the left sided ports prior to beginning the ostomy maturation procedure. A rosebud stoma was then created using standard open technique. The total operative time was 330 minutes with an estimated blood loss of 100 mL.

### 2.2. Postoperative Course

The patient was transferred to the general surgical floor after recovery in the postanesthesia care unit. He was out of bed to a wheelchair on postoperative day two, had return of bowel function (defined as flatus) on postoperative day three, and was tolerating a general diet by postoperative day five. The patient was discharged from the hospital on postoperative day five and did not require any pain medication after discharge from the hospital.

### 2.3. Follow-Up

For the initial weeks following the surgery the patient experienced difficulty initiating urinary drainage from the stoma and Valsalva maneuver was used. By the fourth postoperative week the stoma was draining without difficulty. The initial resistance to urinary flow was speculated to be related to peristomal postoperative edema. By two years postoperatively, the patient reported no urinary tract infections, urethral incontinence, calculi, or high pressure contractions. In addition, a renal ultrasound showed normal kidney parenchyma without hydronephrosis.

### 2.4. Postoperative Urodynamics

Six-month postoperative urodynamic study revealed a stomal leak point pressure of less than 5 cm of H_2_O occurring after 46 mL of infused saline. A repeat urodynamic study performed two years postoperatively showed a stomal leak point pressure of less than 10 cm H_2_O after only 10 mL being infused.

## 3. Discussion

To our knowledge, this is the first robotic-assisted laparoscopic ileovesicostomy performed with a completely intracorporeal bowel anastomosis. Robotic-assisted laparoscopic ileovesicostomy was chosen as the surgical method to manage this patient's neurogenic bladder because it was felt that this method was not only safe and effective, but also provided the patient with the least amount of postoperative complications and fewest risks [10]. In a similar case report describing a pure laparoscopic ileovesicostomy with intracorporeal bowel anastomosis, it was suggested that intracorporeal anastomosis would decrease postoperative ileus by reducing bowel manipulation. The authors also suggested that reduced mesenteric tension would potentially decrease the incidence of ischemia and thrombosis. Furthermore, they proposed that the risk of incisional hernia would be decreased due to the lack of a much larger incision required for an extracorporeal anastomosis [7]. The only study suggesting that bowel anastomosis is not ideally performed intracorporeally is a study by Passerotti and colleagues in which they performed intracorporeal bowel anastomosis on five swine during ileal bladder augmentation [11]. They reported leakage of one bowel-to-bowel anastomosis and determined that it was due to the poor visualization of the backside of the anastomosis during the procedure. However, visualization was excellent in our case and there was no spillage identified (Figure 2). We believe that this theoretical complication can be safely avoided using meticulous robotic surgical technique.

## 4. Conclusion

We report a case of a completely intracorporeal robotic-assisted laparoscopic ileovesicostomy with long term follow-up. The patient in this report continues to have an excellent outcome more than two years postoperatively. Further data is needed to compare robotic-assisted laparoscopic ileovesicostomy outcomes to those performed using a traditional open technique.
